# A Bibliometric Analysis of Corona Pandemic in Social Sciences: A Review of Influential Aspects and Conceptual Structure

**DOI:** 10.1109/ACCESS.2020.3008733

**Published:** 2020-07-13

**Authors:** Adeel Nasir, Kamran Shaukat, Ibrahim A. Hameed, Suhuai Luo, Talha Mahboob Alam, Farhat Iqbal

**Affiliations:** 1 Department of Management SciencesLahore College for Women University 66890 Lahore 54000 Pakistan; 2 School of Electrical Engineering and ComputingThe University of Newcastle 5982 Callaghan NSW 2308 Australia; 3 Punjab University College of Information Technology, University of the Punjab Lahore 54590 Pakistan; 4 Department of ICT and Natural SciencesNorwegian University of Science and Technology 8018 7491 Trondheim Norway; 5 Department of Computer ScienceUniversity of Engineering and Technology 441256 Lahore 54890 Pakistan

**Keywords:** Bibliometric analysis, biblioshiny, conceptual structure, COVID survey, coronavirus, COVID-19, pandemic, r-studio, SARS, social sciences

## Abstract

Corona pandemic has affected the whole world, and it is a highly researched area in biological sciences. As the current pandemic has affected countries socially and economically, the purpose of this bibliometric analysis is to provide a holistic review of the corona pandemic in the field of social sciences. This study aims to highlight significant, influential aspects, research streams, and themes. We have reviewed 395 journal articles related to coronavirus in the field of social sciences from 2003 to 2020. We have deployed ‘biblioshiny’ a web-interface of the ‘bibliometrix 3.0’ package of R-studio to conduct bibliometric analysis and visualization. In the field of social sciences, we have reported influential aspects of coronavirus literature. We have found that the ‘Morbidity and Mortality Weekly Report’ is the top journal. The core article of coronavirus literature is ‘Guidelines for preventing health-care-associated pneumonia’. The most commonly used word, in titles, abstracts, author’s keywords, and keywords plus, is ‘SARS’. Top affiliation is ‘The University of Hong Kong’. Hong Kong is a leading country based on citations, and the USA is on top based on total publications. We have used a conceptual framework to identify potential research streams and themes in coronavirus literature. Four research streams are found by deploying a co-occurrence network. These research streams are ‘Social and economic effects of epidemic disease’, ‘Infectious disease calamities and control‘, ‘Outbreak of COVID 19,’ and ‘Infectious diseases and the role of international organizations’. Finally, a thematic map is used to provide a holistic understanding by dividing significant themes into basic or transversal, emerging or declining, motor, highly developed, but isolated themes. These themes and subthemes have proposed future directions and critical areas of research.

## I. Introduction

**FIGURE 1.**
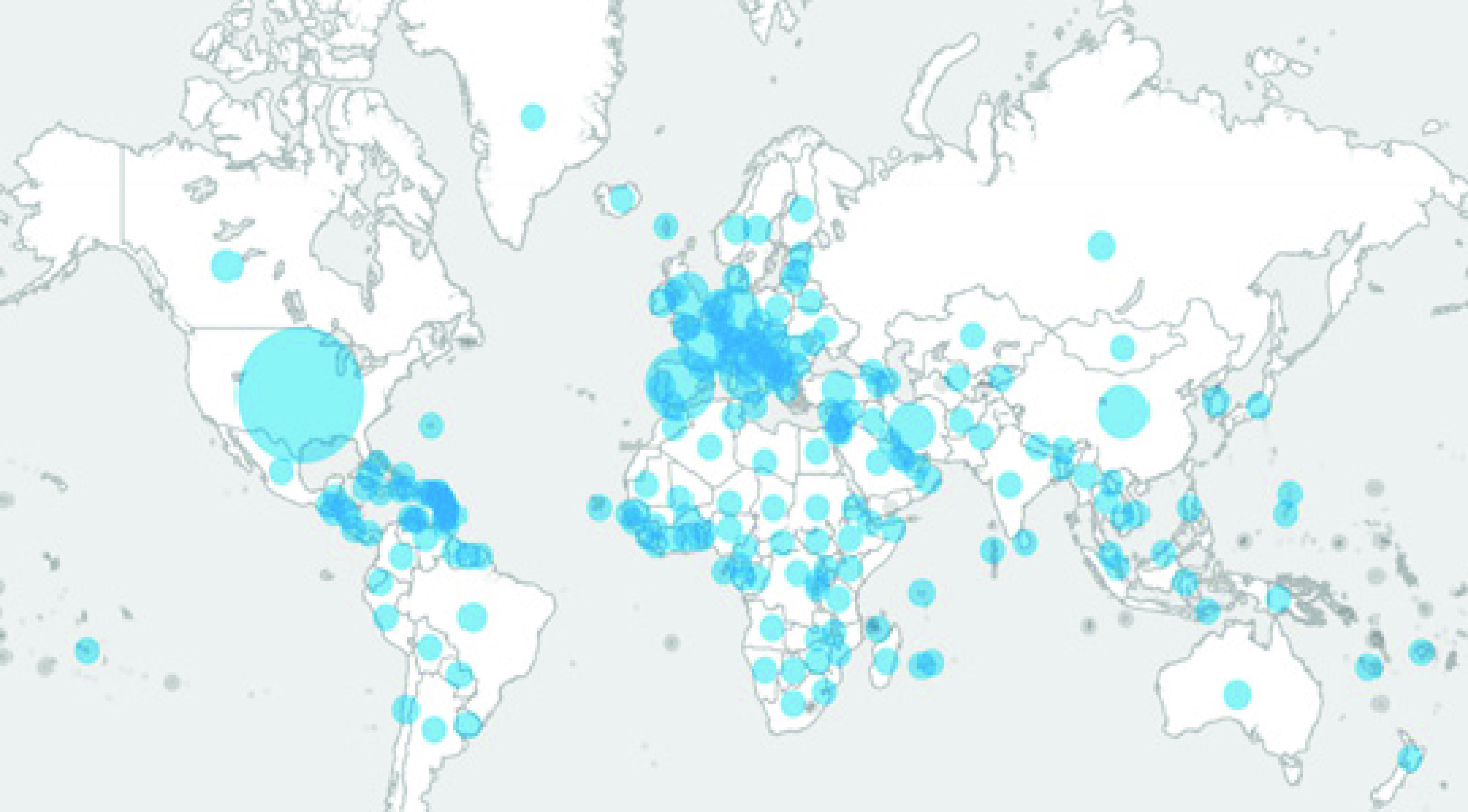
Confirm Cases of COVID-19 in the World [419].

Coronavirus infects humans most commonly leads to Upper Respiratory Infection (URI). It is single-stranded RNA viruses that cause various kinds of illnesses in birds and mammals, including humans. Family of coronaviruses is used to be epidemic, but SARS-CoV-2 is declared as the pandemic by WHO. Figure 1 is a clear representation of the spread of COVID-19. The disease spread from a person with the virus to a person through droplets from the mouth and nose [1]. The impact of SARS-CoV-2 causes social and economic damages throughout the world. Figure 1 shows the daily confirm cases in the world by WHO. As of June 23, 2020, there are approximately 8.86 million confirmed cases across the globe and 0.46 million loss of lives. It has a severe social impact. Moreover, coronavirus has affected the social, business, and economic dynamics of the world. As with COVID-19 outbreak throughout the globe, different governmental pandemic mitigation restrictions (PMR) has been imposed. Under these arrangements, maximum social activities are ban in various countries. Non-essential trade within and outside the states has been restricted. Both the infectious disease outbreak and PMR have a significant effect on the development and growth of society economically [2]. It is essential to propose the solution for socio-economic hindrances created from the outbreak of infectious diseases such as Severe Acute Respiratory Syndrome (SARS), Middle East Respiratory Syndrome (MERS), and SARS-CoV 2.

It is imperative to see that there are various epidemic infectious diseases in the past. These viral diseases have put pressure on societies and economies. This study undertakes the past literature of viral infections related to coronavirus in the field of social sciences, economics, and business and performs a bibliometric analysis. The objective of this research is to analyze the literature and identify top countries, authors, affiliations, sources related to infectious viral diseases. Furthermore, this study aims to find research streams and themes by using the literature of coronavirus in the field of social sciences for the period 2003-2020. The themes and research streams may lead the policymakers, scholars, and researchers to the direction for future research and find answers to current problems. Moreover, it is the quantitative approach to provide a systematic study of written publications.

The bibliometric analysis significantly improves the quality of the literature review by introducing a transparent, systematic, and reproducible review process. It provides means for mapping the research fields and influential work without subjective bias that is imperative for holistic aid to the literature process [3]. For bibliometric analysis, this study uses ‘biblioshiny’ the web-based interface of R-package (‘bibliometrix 3.0’).

## II. The Procedure of Bibliometric Analysis

**FIGURE 2.**
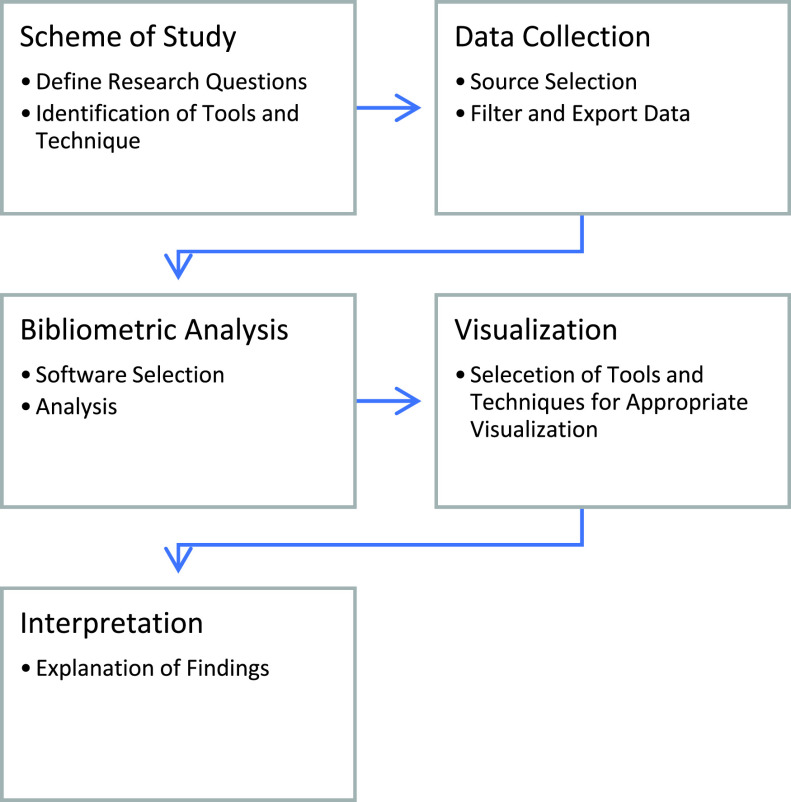
The Procedure of Bibliometric Analysis.

This article proceeds with five steps, known as a bibliometric workflow that is suggested by Zupic and T. Čater [4]. Figure 2 represents the five steps to complete the process of bibliometric analysis of Coronavirus literature.

## III. Scheme of Study

The current situation in the world raises many questions that need to be answered. For this research, we consider the following questions to be answered that will help to identify the dynamics of coronavirus literature and provide holistic means for future research in the field where biological sciences links with social sciences. This study addresses the following questions.

1. What are the influential aspects of coronavirus literature in the field of social sciences?
2. Exploring coronavirus literature of social sciences, what are the main trends and key themes?
3. To prepare for the future, what comprehensive lessons can we take from the past literature, and what future agendas can be set?

We answer question 1 with descriptive analysis and find core sources, authors, countries, publications, and affiliation in the coronavirus publications in the field of social sciences. For core sources and core authors, we have used source impact, total citations (TC), and net publications (NP) per year. Moreover, we identify the core sources by using Bradford’s Law. According to Bradford’s Law, sources are divided into three zones. Zone 1 is highly productive and considered as the nuclear zone. Zone 2 is moderately productive, and sources related to zone 3 has low production [5]. We suggest top countries and affiliations based on publication frequency and total citations.

Core areas of study and key themes are imperative for linking various research streams and generating future direction of study. For this purpose, we have adopted some technical tools such as co-occurrence map, thematic map, and thematic evolution. We use keywords plus for analysis because it describes the knowledge structure of the study and helps to identify and link different research areas [6]. The author’s keywords indicate the main issues of the study. Keywords plus are provided by the database that expresses the article contents succinctly. Keywords plus offer more descriptive trends than the author’s assigned keywords [7]. We are deploying ‘biblioshiny’, which is the bibliometric analysis tool provided by the R-program, to identify research streams and themes using keywords of infectious disease literature.

### A. Objectives, Tools, and Techniques

The objective of this study is to provide the bibliometric review of the literature of coronavirus in social sciences, which can be used to curb the minus created by the infectious disease in the global social network. Moreover, we aim to provide means to improve our economic and social set up under a globalized network from these pathogens. We have subdivided our objective for better understanding.

The first objective of the study is to find core publications, authors, countries, and institutions. To fulfil this objective, we use ‘biblioshiny’, a web-specific R package (‘bibliometrix 3.0’) for descriptive analysis of documents. Tools from the ‘biblioshiny’ interface for analysis are; Bradford’s Law, global citation, h, g, and m-index. The second objective is the find the key research streams and themes. For this objective, we are using science mapping techniques of conceptual structure and using keywords plus as the input data. After completion of objections 1 and 2, we can provide a comprehensive interpretation and define future research agendas.

## IV. Composing of Bibliometric Data

The composition of our bibliometric data has two segments. In the first segment, we select the source from where we can take and analyze articles. For that purpose, we have selected various databases such as Scopus, Web of Science (WOS), Emerald, and google scholar. In the second segment, we have formed the search query for holistic data collection. We have selected the field of social sciences for collection for that purpose, we have applied various filters to our search query and make it match to our objective and optimal results. The final search query is ‘TITLE-ABS-KEY (“coronavirus” OR “corona virus” OR “COVID-19” OR “COVID 19” OR “SARS-CoV 2” OR “betacoronavirus” OR “SARS coronavirus” OR “severe acute respiratory syndrome” OR “MERS-CoV” OR “middle east respiratory syndrome”) AND (LIMIT-TO (SRCTYPE, “j”)) AND (LIMIT-TO (PUBSTAGE, “final”)) AND (LIMIT-TO (DOCTYPE, “ar”)) AND (LIMIT-TO (SUBJAREA, “SOCI”) OR LIMIT-TO (SUBJAREA, “BUSI”) OR LIMIT-TO (SUBJAREA, “ECON”)) AND (EXCLUDE (PUBYEAR, 1999)) AND (LIMIT-TO (LANGUAGE, “English”))’. Approximately all articles are related to a coronavirus search query. The reason for using COVID 19 is to check whether any research hinted towards this keyword in the past. The final search query has able to find 328 articles from social sciences, 27 articles are from economics, econometrics, and finance, and 40 articles are from the field of business, management, and accounting. Business and economics have a significant effect on social wellbeing, which is why we are considering these articles under social sciences. The conceptual framework uses keywords for theme generation, and for efficient analysis, we limit our search query to only journal articles and articles with the English language. The reason for using one comprehensive language to provide efficient bibliometric analysis is that it gives us various tools that compare keywords, articles sources, and affiliations. There are ten articles in other languages, and keyword analysis will perform better in one language. Moreover, we manually analyze the articles, and twelve articles were dropped because they were not according to the objective of this study, our final sample is comprising of 395 articles.

## V. Bibliometric Analysis and Visualization

Bibliometric analysis is the application of statistical and mathematical tools to books and media communication [8]. ‘Biblioshiny’ is the tool under the package which is designed for non-coders to provide means for complete scientometric and bibliometric analysis offering numerous options divided into categories of sources, documents, authors, conceptual structure, social structure, and intellectual structure. It allows obtaining multiple results in the shape of tables and graphs, which are not common in other software [9].

Table 1 provides the descriptive characteristics of corona literature that is imperative to understand before moving forward with the analysis. We finalized 395 documents that only journal articles. All these journals use 1699 keywords plus and 873 author keywords. The period we used for corona literature is from 2004 to 2020.

**TABLE 1:** Descriptive Characteristics of Corona Literature

Description	Results
Documents	395
Sources (Journals)	243
Keywords Plus (ID)	1699
Author’s Keywords (DE)	873
Period	2003 - 2020
Average citations per documents	16.18
Authors	1492
Author Appearances	1671
Authors of single-authored documents	104
Authors of multi-authored documents	1388
Single-authored documents	117
Documents per Author	0.265
Authors per Document	3.78
Co-Authors per Documents	4.23
Collaboration Index	4.99

A total of 1492 authors wrote these documents; among them, only 104 articles are with a single author. There is a high collaboration in corona publications that is shown by the collaboration index. Document per author ratio is 0.264, which means, on average, almost four authors have written one document.

**FIGURE 3.**
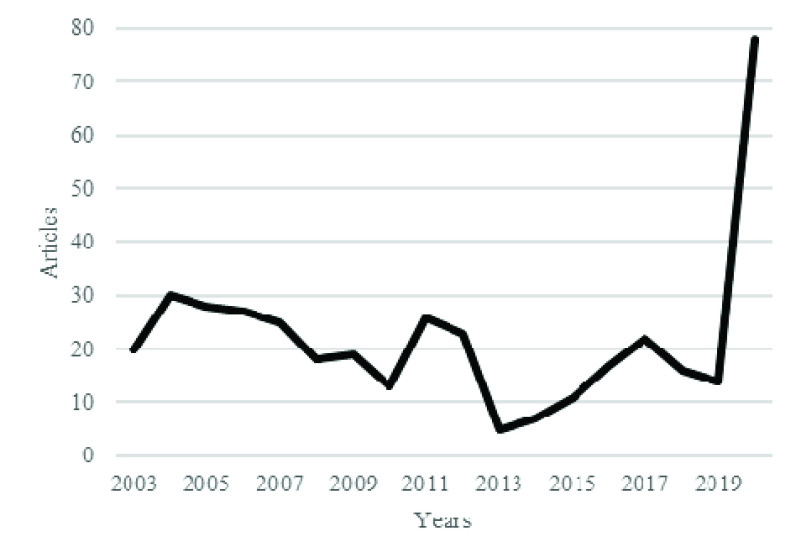
Annual scientific production.

**FIGURE 4.**
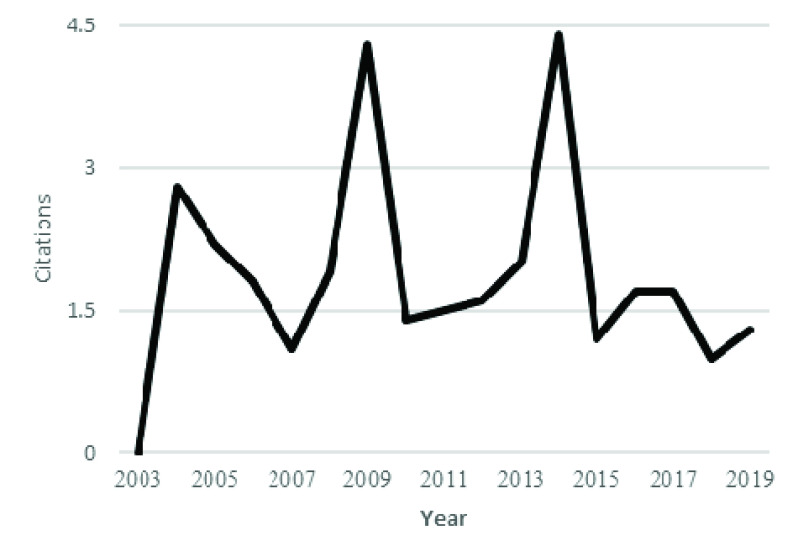
Average article citation per year.

Figure 3 shows the annual production, and figure 4 shows the citation per year for coronavirus publications. There is limited production at the start, but literature production increases with time, especially after the identification of Severe Acute Respiratory Syndrome (SARS) coronavirus in the Guandong province of China. SARS was also an epidemic disease affecting 26 countries and resulted in more than 8000 cases. At that time, implementations of feasible practices for infection control brought the end to this infection [10]. After the control, there was a decrease in both production and citation of coronavirus studies. However, then again, after 2010, there was an increasing trend in the publications and citations of coronavirus studies. There was a significant increase in the annual citation of coronavirus publications from 2013 to 2015. This increase in citation and total production after 2015 is due to the outbreak of MERS.

**FIGURE 5.**
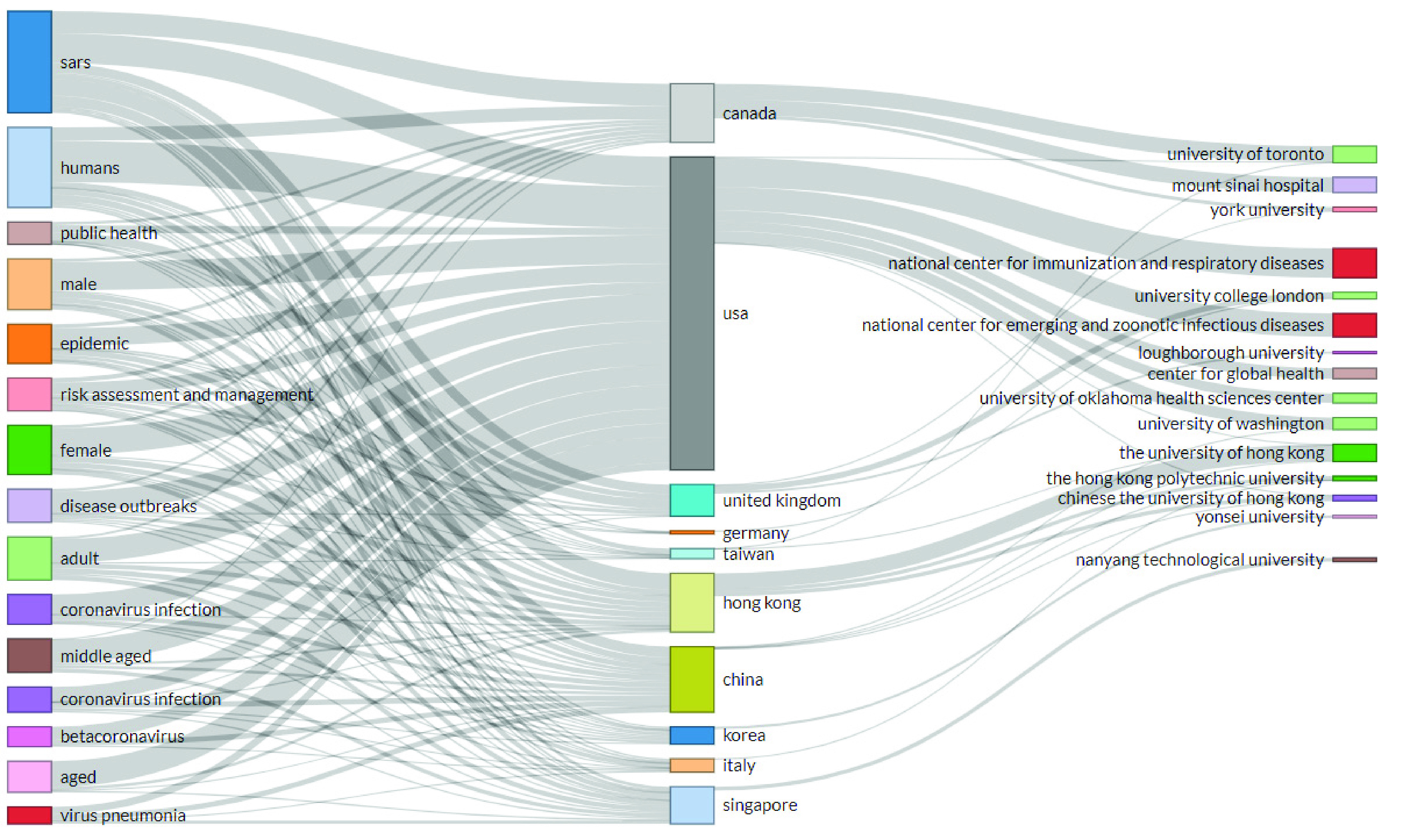
Three-fold analysis of corona literature.

In addition to the annual production and article citations per year, it is imperative to see the main topics, places, and affiliations of coronavirus publications. Figure 5 presents the three-fold analysis of coronavirus publication with a keyword plus on the left side of the figure, affiliations on the right, and countries of interest in the middle. The figure shows that the USA is working with most of the top affiliations concerning topics related to the outbreak of infectious disease. Furthermore, China, Hong Kong, Canada, and the United Kingdom have significant contributions based on various social science topics of infectious disease. Issues related to SARS are most studied in most of the countries.

### A. Influential Aspects of Coronavirus Literature

#### 1) Core Journals

To find the core journals, involved in publishing coronavirus literature in the field of social sciences, we use source impact and Bradford Law. Table 2 ranks the articles based on h, m, g-index, total citation (TC), and net production (NP) and publication starting year (PY_start). Table 3 represents Bradford law, which divides the journal into three zones. Zones 1 represents core sources for publishing coronavirus articles. This is the nuclear zone representing journals having significant publications. Table 3 reports the journal rankings according to Bradford law. We have found that out of 395 journals, 23 journals comes under core zone 1, the rest of the journals are in zone 2 and zone 3. Top 23 journals are the core publishing sources for corona literature in social sciences.

**TABLE 2:** Top Ten Journals According to Source Impact

Top 10 Journals	h_index	g_index	m_index	TC	NP	PY_start
Morbidity and Mortality Weekly Report	11	18	0.647	342	33	2004
Social Science and Medicine	9	9	0.529	670	9	2004
Health Security	4	6	0.800	36	8	2016
Building and Environment	7	7	0.438	640	7	2005
Economic and Political Weekly	0	0	0.000	0	6	2020
Fortune	1	1	0.056	4	6	2003
Tourism Management	5	6	0.333	401	6	2006
Asian Journal of Social Psychology	4	5	0.235	107	5	2004
Bioscience Trends	4	5	0.333	109	5	2009
International Journal of Hospitality Management	5	5	0.278	217	5	2003

**TABLE 3:** Journal Rankings According to Bradford Law

Sources	Rank	Freq	cumFreq	Zones
Morbidity and Mortality Weekly Report	1	33	33	Zone 1
Social Science and Medicine	2	9	42	Zone 1
Health Security	3	8	50	Zone 1
Building and Environment	4	7	57	Zone 1
Economic and Political Weekly	5	6	63	Zone 1
Fortune	6	6	69	Zone 1
Tourism Management	7	6	75	Zone 1
Asian Journal of Social Psychology	8	5	80	Zone 1
Bioscience Trends	9	5	85	Zone 1
International Journal of Hospitality Management	10	5	90	Zone 1
Asian Journal of Communication	11	4	94	Zone 1
Journal of Contingencies and Crisis Management	12	4	98	Zone 1
Scientometrics	13	4	102	Zone 1
Sustainability (Switzerland)	14	4	106	Zone 1
Asia Pacific Journal of Tourism Research	15	3	109	Zone 1
Australian Journal of International Affairs	16	3	112	Zone 1
Biosecurity and Bioterrorism: Biodefense Strategy, Practice, and Science	17	3	115	Zone 1
Current Issues in Tourism	18	3	118	Zone 1
Eurasian Geography and Economics	19	3	121	Zone 1
International Journal of Human Resource Management	20	3	124	Zone 1
Journal of Chemical Information and Modelling	21	3	127	Zone 1
Journal of Health Communication	22	3	130	Zone 1
Medical Teacher	23	3	133	Zone 1
Medicine and Law	24	3	136	Zone 2
Safety Science	25	3	139	Zone 2

Morbidity and mortality weekly report (MMWR) is a significant platform for publishing coronavirus literature in the field of social sciences. From its recent publications by Ng *et al.* in [11] stating that during COVID-19 pandemic, test the multipronged surveillance system, in which they provide means to detect coronavirus patients in Singapore. Furthermore, the reporting provided by Burke *et al.* in [12] and Jernigan in [13], indicated the effect of COVID-19 in the United States and suggested monetary measure to the center of disease control, states, local and public health care to reduce the spread of COVID-19. 70% of symptomatic persons have a travel-related risk of getting coronavirus, 20% having contact with laboratory diagnosed patients. 9% had both risk factors [14].

Published in MMWR, As reported by Foote *et al.* in [15], the urgency of Isolation and rapid recognition during the infectious disease outbreaks. The study used unannounced mystery patient drills in 49 New York City hospital emergency departments to test protocols and the ability of staff members to manage and identify the infectious patients. The results of the drills indicated that infectious patients were masked and isolated 78% of drills. Moreover, 88% of the time, patients were masked and isolated when travel history was obtained, and 21% when it was not. Median time taken by staff for masking was 1.5 minutes and 8.5 minutes for isolation. The study suggested tool kits for similar drills to enhance the health care system in the United States. The nosocomial outbreak of SARS infectious disease in the health care hospital in Riyadh is also covered by MMWR [16]. It has also published reports regarding found cases and has suggested public guidelines about the Middle-East respiratory syndrome coronavirus (MERS-COV) infection [17]–[19].

**FIGURE 6.**
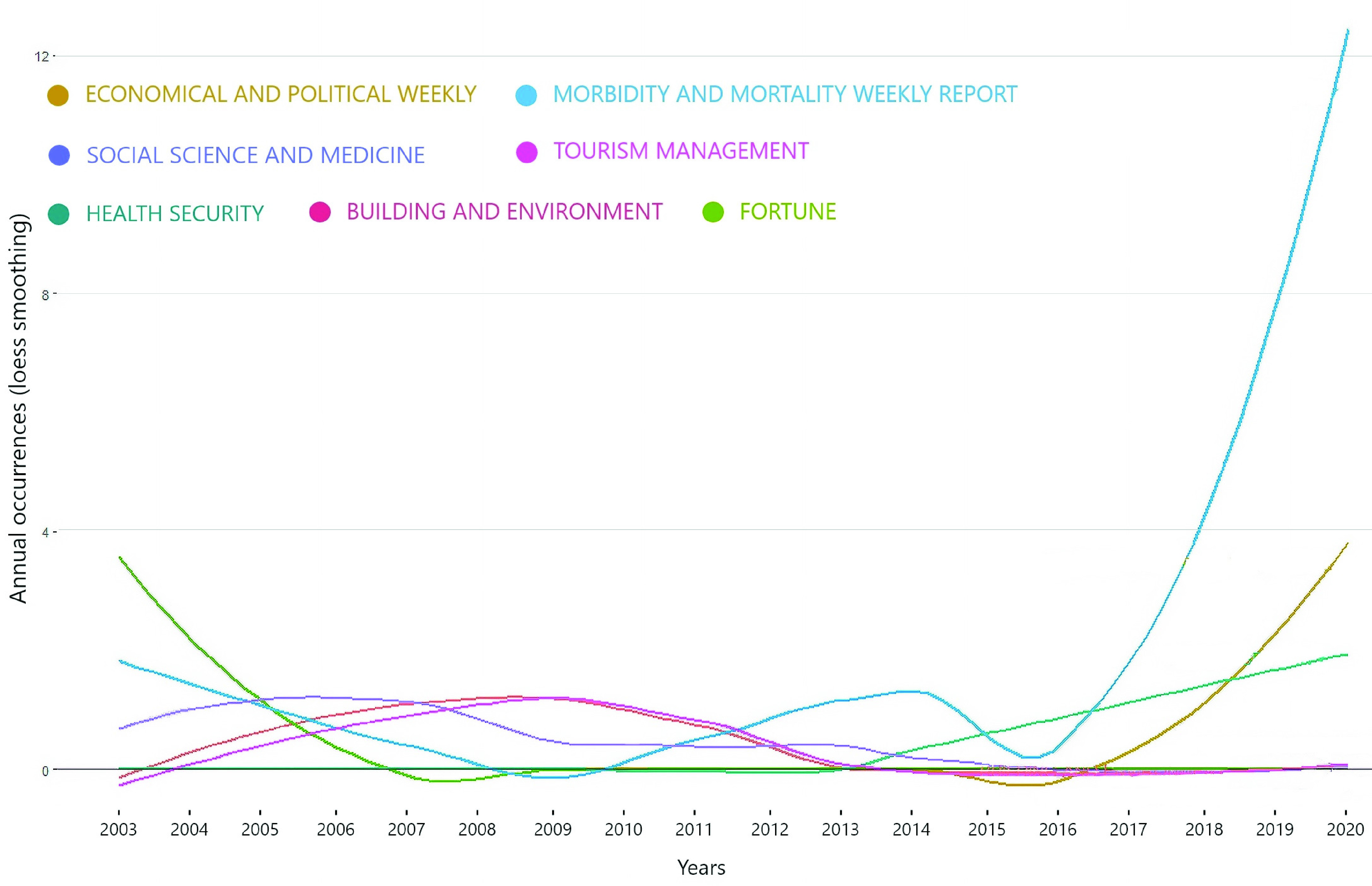
Source Growth.

Figure 6 shows the growth in the publication by top journals. We use the loess smoothing technique that is the locally weighted smoothing use regression analysis to demonstrate the smooth line with the help of a time plot or scatter plot. Loess smoothing helps to understand treads through time [20]. There has been significant growth in publications by MMWR since 2010. it represents the primary source of coronavirus literature in social sciences. Economic and political weekly have a decreasing trend from 2014 to 2016, then there is a Sharpe increase in the publications from 2016 onwards. Health security has a significant growth for the past three years. Scholars and policymakers should go through these publications during pandemic times because these journals can provide useful information for infectious diseases their socio-economic impact. The rest of the journals has no significant loess smoothing when regress through time.

#### 2) Core Journal Articles

This section highlights the leading articles in coronavirus publications in the field of social sciences. We have listed down the top 10 globally cited articles in table 4.

**TABLE 4:** Most Globally Cited Article

Paper	Total Citations	TC per Year
Guidelines for preventing health-care-associated pneumonia [21]	986	58.00
Policies and technical guidelines for urban planning of high-density cities - air ventilation assessment (AVA) of Hong Kong [22]	248	20.67
Health care workers’ ability and willingness to report to duty during catastrophic disasters [23]	241	15.06
Responding to global infectious disease outbreaks: Lessons from SARS on the role of risk perception, communication, and management [24]	201	13.40
Perceived travel risks regarding terrorism and disease: The case of Thailand [25]	148	12.33
Distinguishing knowledge-sharing, knowledge-construction, and knowledge-creation discourses [26]	138	11.50
Disease metaphors in new epidemics: The UK media framing of the 2003 SARS epidemic [27]	119	7.44
The impact of crisis events and macroeconomic activity on Taiwan’s international inbound tourism demand [28]	114	9.50
Representations of SARS in the British newspapers [29]	98	5.76
The airborne transmission of infection between flats in high-rise residential buildings: Particle simulation [30]	95	7.31

On top of the list, Tablan *et al.* [21] conducted the study in 2004 regarding guidelines for reducing the incidence of severe acute respiratory infections in health care settings and acute care hospitals. The study from Ng [22] is second on the list of most globally cited articles and can be used for future remedial research regarding coronavirus impacts on the globalized world. They investigated the spread of SARS in 2003 in Hong Kong.

Ng in [22] discussed the implementation and scientific process of air ventilation assessment (AVA) system in urban areas of Hong Kong. Qureshi in [23] reported the surge capacity needs in the situation of catastrophic disasters. He has surveyed surge capacity from health workers and found that 83% were willing to report in the circumstances of mass casualty incidents, but 64% were willing to report in the situation of the SARS outbreak. He also identified that barriers to willingness were fear and concern for family and oneself and tentative health problems.

Smith [24] highlighted the main concerns for the future from the outbreak of SARS in 2006 that we are facing in the 2019 pandemic. He indicated that the epidemic of SARS might assist in dealing with the pandemic situation in the future. He suggested that perception of risk, management, and communication played an essential role in the economic impact of infectious disease. Furthermore, the role of official organizations and media and the establishment of risk priorities for a better system might prevent a future global pandemic. We do not agree with his conclusion that there is low potential for the spread of infectious disease after SARS.

Rittichainuwat and Chakraborty [25] investigated the impact of infectious diseases such as SARS into the hospitality industry of Thailand. Their article comes on fifth highly cited article among 395 articles. They suggested that travelers continue traveling in the time of crises, but they choose less dangerous destinations.

#### 3) Core Words

Table 5 provides the most frequent words used in coronavirus literature in the field of social sciences. The table is divided into four parts of keywords plus, Authors Keywords, Abstract, and Title.

**TABLE 5:** Most Frequent Words

Keyword Plus		Author Keyword	
Words	Occurrences	Words	Occurrences
sars	228	sars	55
humans	158	covid 19	30
epidemic	85	severe acute respiratory syndrome sars	18
male	83	Hong Kong	17
female	77	china	16
adult	72	crisis	11
virus pneumonia	60	pandemic	11
risk assessment and management	59	coronavirus	9
coronavirus infection	58	epidemics	9
disease outbreaks	58	influenza	9
Abstract		Title	
Words	Occurrences	Words	Occurrences
health	464	sars	100
sars	446	health	67
public	264	COVID	51
respiratory	248	respiratory	51
outbreak	239	syndrome	43
disease	230	outbreak	37
syndrome	221	public	35
severe	211	crisis	33
study	207	disease	33
acute	195	coronavirus	29

In all parts, SARS is the most common word. Social sciences are related to society, economy, and people, which has been shown in the keywords as well. These keywords are related to infectious diseases, public health, humans. In coronavirus literature, minimal author keywords are used. Keyword plus covers the broad topics, where there are common infectious diseases used as keywords. Additionally, keywords related to Human, male, female, and middle-aged indicate the relationship between coronavirus and society.

**FIGURE 7.**
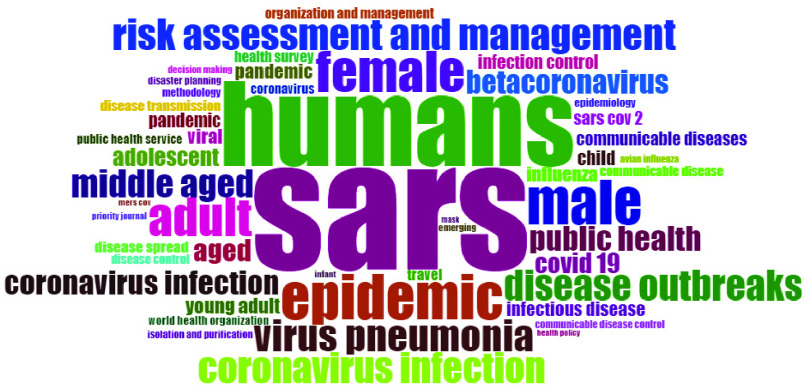
Word Cloud.

There are topics related to disease outbreak and epidemic. Not many researchers used author keywords. Health is the most used word in the abstract, and SARS is the highly used word in the title. Terms used in abstract and title are more generic that are less likely to produce any theme or research stream. Figure 7 shows the word cloud made from keyword plus. Words with high frequency in the literature are more in size. Humans and SARS have the highest frequency in the literature of social sciences. So, these are the biggest of all the words used. Then there is an epidemic, which has a strong history in China and Canada. The literature is about social sciences. That is why much research is done on the social aspects of society. Like many researchers link the infectious disease with humans, gender, age groups animals, etc. There are studies on communicable diseases, disease outbreaks, and viral infections. Some studies link the infectious diseases with travel history, so travel is used as a keyword. Isolation and purification are used in coronavirus literature as a keyword, and it is crucial as well because it helps to strengthen the isolation strategies further and secure the society. Risk assessment and infection control are also used as the keyword in literature, and these studies can be used to elaborate on the solutions for epidemic control further. All these keywords are linked and address many problems that the world is facing today. Use this literature and find a solution that can save the globalized economies from a possible future pandemic.

**FIGURE 8.**
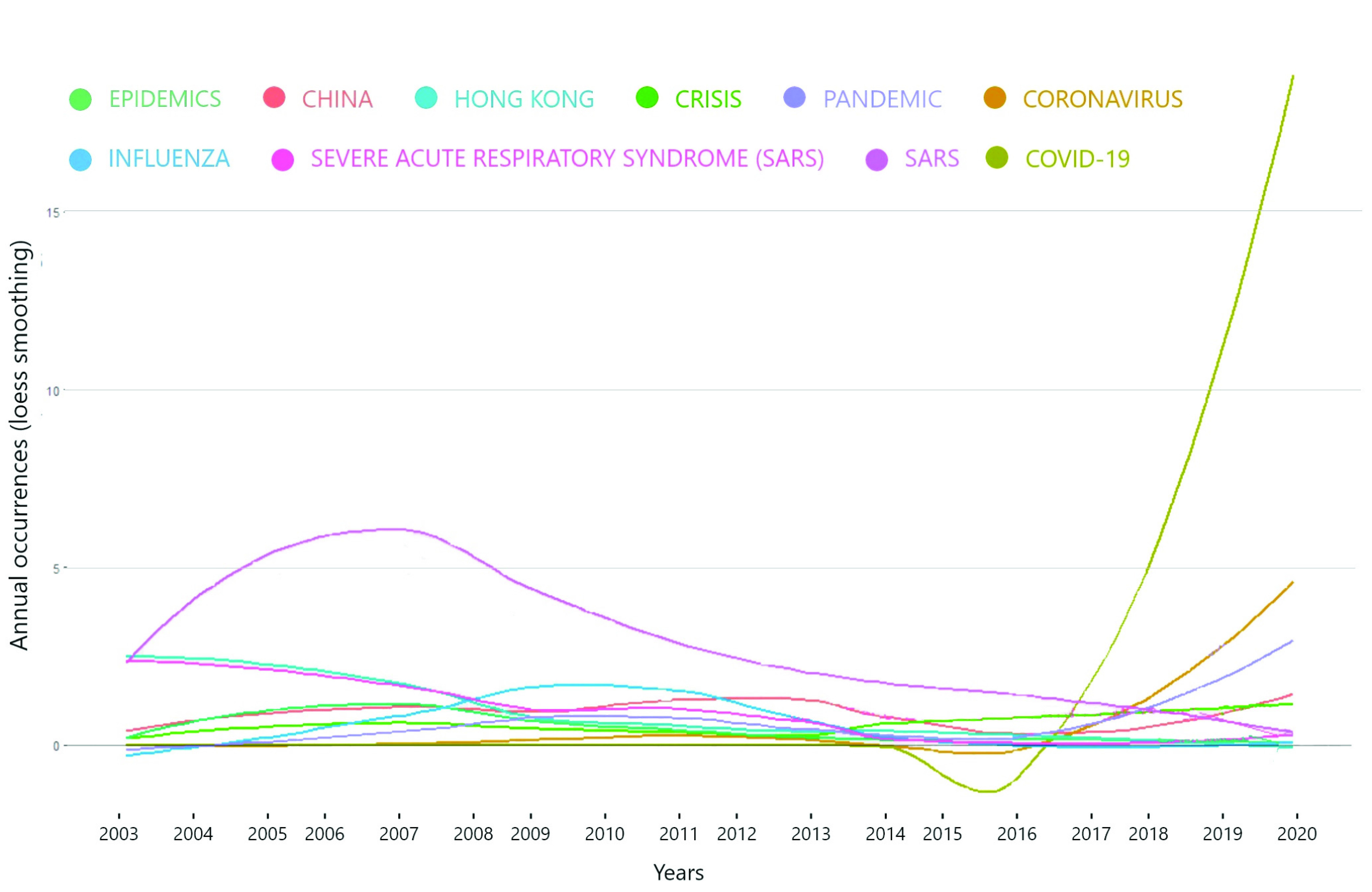
Word Growth Overtime.

In addition to the word cloud, figure 8 gives us a picture of word growth in literature through time. As shown in the figure, the coronavirus keyword started to grow after 2010. The body of knowledge began to identify the need for this problem to get solved. Beta coronavirus represents coronaviruses one of four genera that infect bets mainly, but the infection can be found in other species like camels, rabbits and humans [31]. Figure 8 uses a loess smoothing technique to analysis the growth of keywords usage overtime. The use of COVID 19 keyword decreases after 2014, but after 2017, there has been a Sharpe increase in the topics related to COVID 19. Throughout the years from 2000 to 2013, SARS dominated the rest of the keywords, but after 2013 there is a decrease in SARS keyword and increase in coronavirus related topics. The growth has been observed in keywords such as coronavirus, pandemic and china.

#### 4) Main Authors, Affiliation, Institutions and Countries

This section provides information regarding core authors, affiliations, institutions and countries of coronavirus literation in the field of social sciences. There are ten authors having a more significant impact in coronavirus literature, and these authors are listed in table 7. Ranking is on the basis of h-index.

**TABLE 6:** Top 10 Authors Impact in Corona Pandemic Literature

Author	h_index	g_index	m_index	TC	NP	PY_start
Chau	4	4	0.235	91	4	2004
Ho	4	4	0.235	91	4	2004
Ali	3	4	0.188	89	4	2005
Huang	3	4	0.273	69	4	2010
Gerber	3	3	0.429	97	3	2014
Li	3	3	0.176	85	3	2004
Rha	3	3	0.375	30	3	2013
Smith	3	3	0.200	256	3	2006
Watson	3	3	0.429	97	3	2014
Zhang	3	3	0.176	28	3	2004

**TABLE 7:** Top Countries in Terms of Publications and Citations

Country and Regions	Frequency	Country and Regions	Total Citations
USA	236	Hong Kong	1291
Canada	79	USA	877
China	75	UK	568
UK	52	China	453
Taiwan	45	Taiwan	444
South Korea	31	Canada	379
Singapore	26	Thailand	148
Germany	18	Singapore	98
Italy	16	Australia	97
Australia	15	Korea	65

Chau is at number one on the list of authors with the highest impact. Chau, with collaboration with Ho (ranked second), published profoundly impact journal articles. Wong *et al.* [32] discussed environmental quality and human health. They indicated that despite the absence of the central ventilation system, the nasal discomfort was the primary source of sick building syndrome (SBS). Moreover, they surveyed about indoor environmental quality (IEQ) and concluded that noise rather ventilation was the major IEQ problem. They, in [33], analyzed the impact of crises shock on housing prices using the hedonic price model. They reported a negative impact of SARS on housing prices. Just before this article, they in [34] observed a similar impact on the town planning board (TBT) and housing developers. They concluded that TBT was effected by the shocks created from the outbreak of infectious disease, but developers were not affected by any shocks. They, in [35], investigated the stigmatization created from SARS. They indicated that many residents were affected by stigma in various sorts of being insulted, shunned, and rejected in the domain of work, schooling, services, and interpersonal relationships. According to their respondents, stigmatization decreased but never disappeared after the outbreak.

Ali (Third rank) in [36] discussed the disease outbreak in a political and social context. He suggested that infectious disease outbreaks should be addressed from a global perspective. Ali *et al.* [37] and Ali and Keil [38] discussed the risk of SARS and its mitigation. He described the political, economic, and social risk factors created from the pandemic and implications of these risk factors in a globalized world. Furthermore, Sanford and Ali [39] discussed various challenges for public health in Toronto after the SARS outbreak.

Huang (ranked four) in his single-authored manuscript [40] described the deteriorated seafood market of china because of the drug residue incidents. He stated the food safety policies and state control over the animal epidemic after the SARS outbreak. He further disclosed through his findings that the state faced hindrance local formers for transforming public rural vets into technology savvy and market-sensitive professionals that can discipline the fish market. His second article discussed the model for individual vulnerability and challenges from the outbreak of an infectious disease such as SARS and H1N1 [41].

Gerber is number fifth on the list of authors, but he has contributed significantly to the field of SARS, MERS, and social sciences. He has started publishing from 2014 with three net publications. He has 97 total citations. Gerber’s first two articles were published in MMWR that studied the first cases of MERS-COV identified in the United States and highlighted the guidelines for health authorities, clinicians, and the public [17], [18].

Li (rank sixth) studies the transmission of viruses and bacteria in [42]. The result of the study suggested that droplets and particles of virus transport short distance during breathing, but it traveled to longer distances during cough. Rha (ranked sixth) in [19] reported SARS as a novel coronavirus in the United States. Further, Rha reported the growth of MERS-CoV infection in the United States and provided guidelines for public health authorities, clinicians, and the general public [17]. Smith is on number eight concerning h and g-index, but he possesses the highest number of citations. Smith, in [24], discussed the risk of a more lethal outbreak than SARS in 2003. He suggested how people perceive the risk of disease outbreak was effected by the role of official organizational policies, research priorities, and the role of media. Smith, in [43], analyzed the economic impact due to the outbreak of infectious diseases. The study suggested that due to pandemic situations, specific steps were taken by states such as school closure and absenteeism from work. The authors suggested that pandemic became the reason for 0.5% to 2.0% GCP loss. In the case of school closure and absenteeism from work, this impact became triple in size.

Table 7 shows two sets of data; the left side represents the countries and regions with many scientific productions over time. On the right side, there are countries with several citations. As the USA is on top with several publications, Canada ranked second, and china ranked 3^rd^, but in citations, Hong Kong’s total citations are better than the USA. The USA, in the second position, when it comes to citations. The UK published 52 articles on infectious disease in social sciences, but they have 568 citations. Chine comes on forth in terms of citations.

**FIGURE 9.**
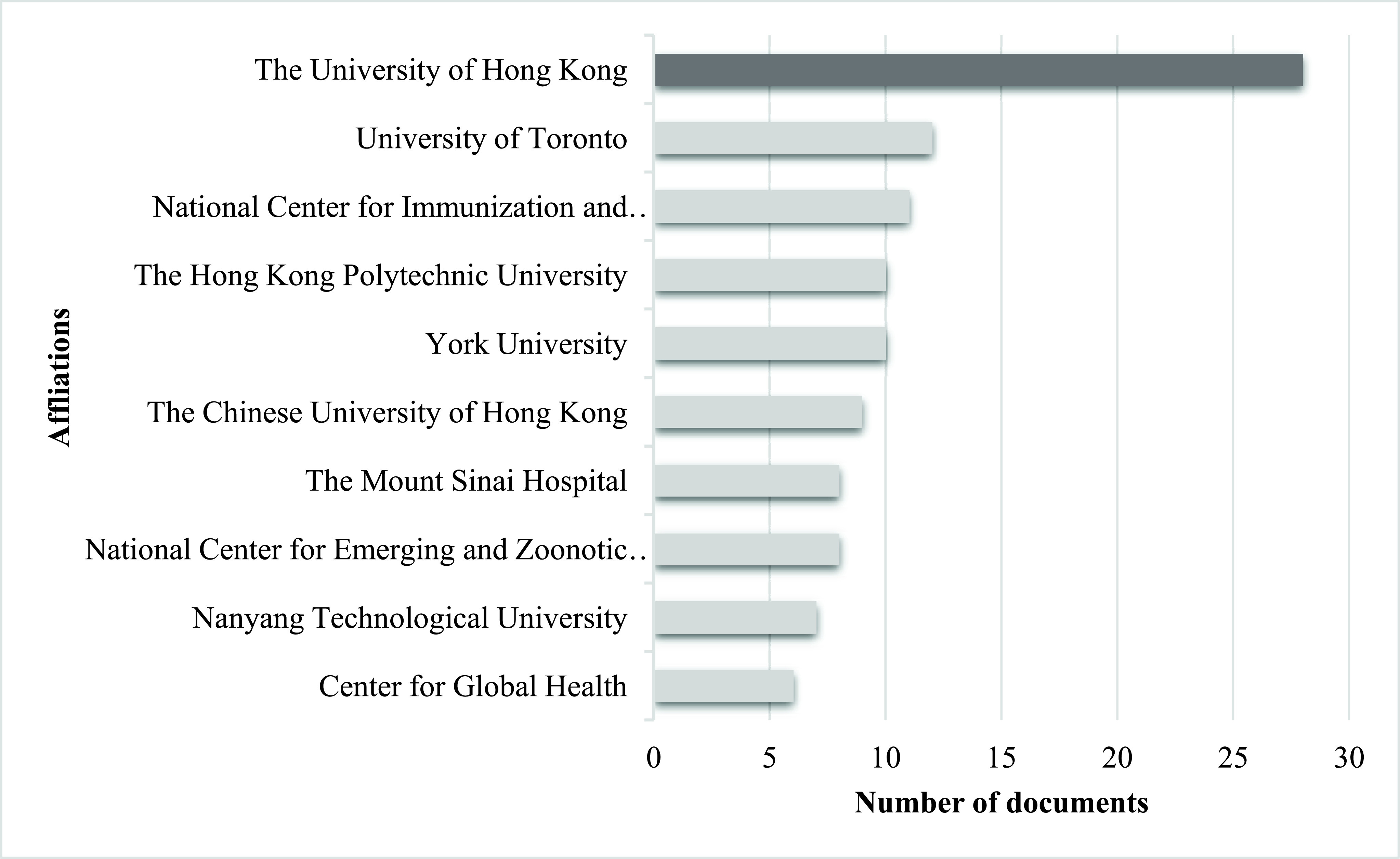
Most Relevant Affiliations.

Most relevant affiliations are reported in figure 9. The University of Hong Kong (HKU) comes in the first place. The university has provided a strong basis for research on infectious diseases. It has started the HKU forum on the COVID 19 pandemic and HKU research in COVID 19 for quality publications [44]. University of Toronto (UT) comes on the second list of most relevant affiliations. UT founded in 1827 and became the leading institution of Canada for knowledge creation, discovery, and learning. UT is Canada’s leading research institution [45].

The national center for immunization and respiratory diseases (NCIRD) at third the list of affiliations. NCIRD is responsible for coordinating, planning, and conducting immunization operations in the United States. It is the part of centers of disease control and prevention. NCIRD provides training, consultation, educational, epidemiological, statistical, and promotional services to state and local health departments in the US [46]. On the fourth position, The Hong Kong Polytechnique University is 80 years old research university. They solve real-world challenges with their diverse interdisciplinary research [47]. Yorks University offers a significant research base. With 95 million in research grants and awards, number 1 in global joint research publications, and 25 research centers, Yorks University comes 5^th^ on the list of impactful affiliations [48]. The Chinese University of Hong Kong comes in sixth place as the impactful affiliations in the publication of infectious disease. It was founded in 1963 with a comprehensive researching university [49]. ‘Another day and another breakthrough’ is the slogan of the research center of Mount Sanai Hospital (ranked seventh). It like a technological and scientific revolution with the mission to create advanced treatments for patients through research laboratories and multi-disciplinary centers [50].

The national center for emerging and zoonotic infectious diseases (NCEZID) is eight on the list of most relevant affiliations. There are seven divisions of NCEZID that work with partners in the United States and all around to world for prevention of illness and deaths from various infectious diseases. Their objective is to protect people from health threats such as foodborne and waterborne illness, and infections spread in hospitals, antibiotic-resistant infections, a deadly disease like Ebola and anthrax, an illness that affects travelers, the disease caused by contact with animals such as SARS, MERS and corona and diseases spread by fleas and mosquitoes [51]. Center of global health (CGH) provides its unique scientific knowledge, technical skills, and research to take global public health actions. Its mission is to improve the safety, security, and health of Americans while restricting mortality and morbidity worldwide [51]. It is the 10^th^ most relevant affiliation for coronavirus publications.

Table 8 presents the data regarding the top 10 corresponding author countries, and the USA is at number one. Corresponding authors from the USA have published 64 articles from which 48 are single country publications (SCP), and 16 are multiple country publications (MCP).

**TABLE 8:** Corresponding Author’s Country

Countries and Regions	Articles	Freq	SCP	MCP	MCPratio
USA	64	0.24	48	16	0.25
Hong Kong	44	0.17	33	11	0.25
Canada	27	0.10	22	5	0.185
China	21	0.08	16	5	0.238
Taiwan	19	0.07	15	4	0.211
Korea	14	0.05	12	2	0.143
United Kingdom	14	0.05	9	5	0.357
Singapore	9	0.03	8	1	0.111
Italy	7	0.03	5	2	0.286
Australia	6	0.02	5	1	0.167

Multiple country publications refer to at least one co-author from a foreign country. Hong Kong is in the second position with correspondence of 44 articles, with 33 SCP and 11 MCP. There are 27 publications in Canada with 22 SCP and 5 MCP. China is the fourth position with the correspondence of 21 research articles for infectious diseases. It has SCP of 16 and MCP if 4. Taiwan comes in the fifth position with 19 corresponding research articles.

The issue of coronavirus is related to global social networks, and for the safety of diverse societies, major collaborations are required on coronavirus literature in the field of social sciences. Table 9 has addressed this issue, but unfortunately, very little collaboration has been observed with countries around the globe. China collaborated with the USA in 3 articles, and the USA collaborated with Korea in 5 publications. The rest of the countries in the table collaborated in 4, 3, and 2 articles only.

**TABLE 9:** Collaboration Network

From	To	Frequency
China	USA	9
USA	Korea	5
UK	Germany	4
UK	USA	4
USA	Canada	4
USA	Taiwan	4
UK	Hong Kong	3
USA	Hong Kong	3
Australia	Korea	2
China	Hong Kong	2

**TABLE 10:** Keywords in Each Cluster

Keywords	Clusters	Research Stream
Public health, uncertainty, unemployment, SARS, epidemic, hotel industry, Asian economy, China, human resource management (HRM), labor market, service sector	Red	Social and economic effects of epidemic disease
SARS, crises, resilience, infectious disease, quarantine, tourism, crisis management, tourism demand, globalization, influenza	Blue	Infectious disease calamities and control
COVID 19, coronavirus, SARS-CoV 2, pandemic, social media, epidemic	Green	The outbreak of COVID 19
Infectious diseases, global health governance, avian influenza, international health organizations, world health organization	Purple	Infectious diseases and the role of international organizations

**TABLE 11:** Themes and Keywords in Thematic Map

Cluster Representation	Theme	Keywords in Clusters
SARS	Basic Theme	SARS, risk assessment and management, public health, infectious disease, disease spread, disease transmission, disease control, world health organization
Humans	Basic Theme	Humans, epidemic, disease outbreak, coronavirus infection, male, female, adult, middle age, pandemic, viral
Communicable disease	Emerging theme	Communicable disease, organization and management, health survey, methodology, emerging, population surveillance, public health administration
Infection control	Motor theme	Infection control, public health service, education, psychological aspect, attitude
Avian influenza	Highly developed and Isolated Themes	Avian influenza, health care personal, attitude, acquired immune deficiency syndrome
Coronavirus	Highly developed and Isolated Themes	Coronavirus, respiratory disease, viral disease, computer simulation

### B. Conceptual Framework

This section helps us to understand various themes using the relationship among words (keyword plus). At first, the study proposes a co-occurrence network that allows us to evaluate multiple topics of coronavirus pandemic in social sciences over time. Then we will put these networks of words on a bi-dimensional matrix called ‘Thematic Map’ to analyze to centrality and density of the network.

#### 1) Co-Occurrence Network

**FIGURE 10.**
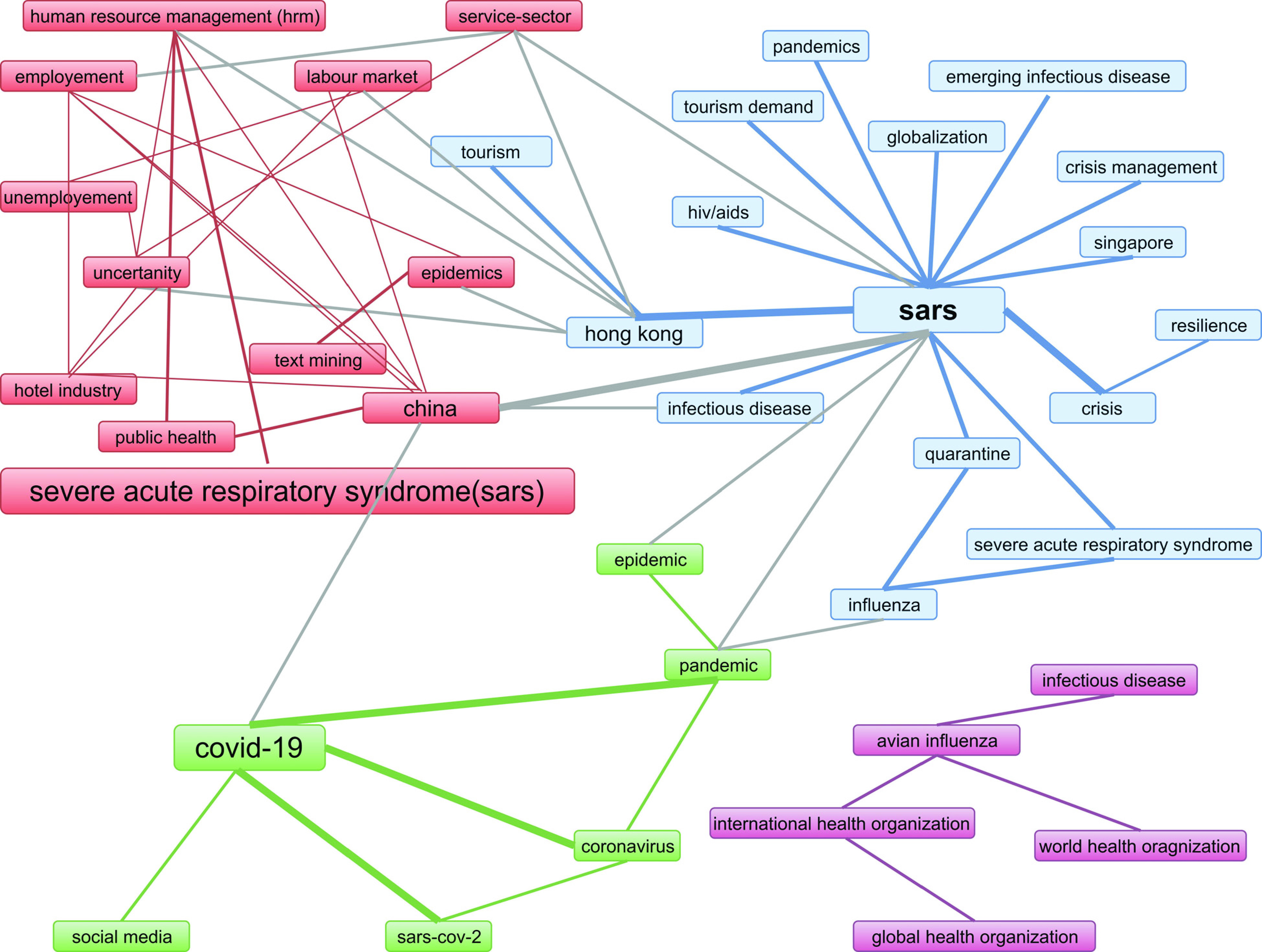
Co-occurrence network.

Figure 10 shows the co-occurrence network of keyword plus. The figure is extracted from the ‘biblioshiny’ of R-package (‘bibliometrix’). The co-occurrence network of keywords is showing that coronavirus literature in social sciences can be divided into four streams. Red cluster is the central cluster with high centrality, blue and green clusters are linked together in terms of themes. SARS coronavirus has the highest centrality. Each group divides the coronavirus literature in various research streams.

The red cluster represents the main coronavirus and discusses multiple characteristics of the viral disease. Its epidemic effect on humans and how it outbreaks and communicate from person to person. This research stream is named as ‘social and economic effects of epidemic disease’. The blue cluster represents the research stream of ‘Infectious disease calamities and control’. In this research streams, studies are related to the identification of risk factors from disease outbreaks, health care policy and knowledge about the disease, precautions among people and controlling authorities, preparations to tackle infectious disease, practices, and attitudes of people and government towards contagious disease. The green cluster represents the research stream of the medical solution to contemporary viral disease, i.e., COVID 19. It describes various tests, methodologies, and cures to curb the effect of epidemic disease. It also covers the topics related to the role of social media in communicating and educating the society about novel coronavirus. Purple cluster is the isolated cluster, yet it the important one. It relates to the crucial role of international health organizations in controlling and managing the spread of infectious disease. The purple research stream is titled ‘Infectious disease and the role of international organizations’.

#### 2) Thematic Map

**FIGURE 11.**
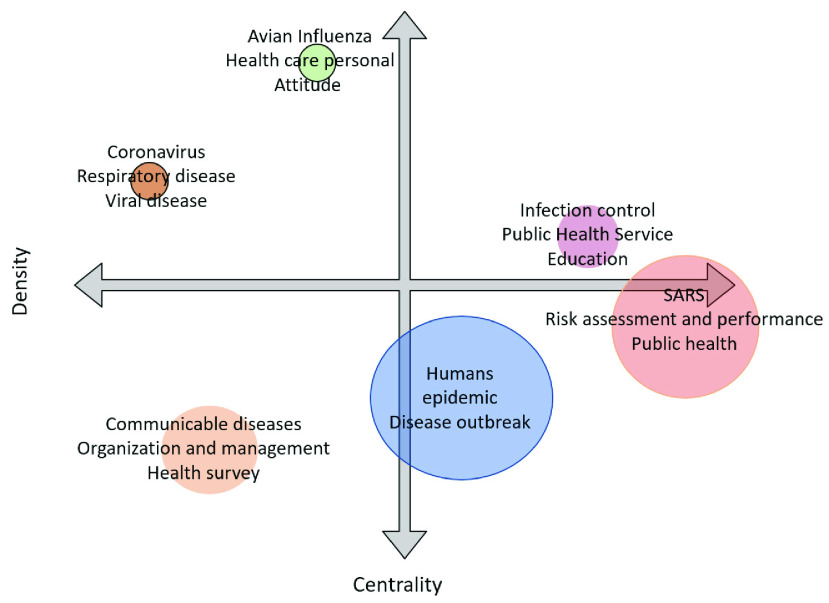
Thematic Map.

We have detected some research themes now for the superior interpretation of the results. We can categories the identified themes into a strategic diagram to analyze the importance and development of the research theme [52]. Figure 11 represents the thematic map based on density (y-axis) and centrality (x-axis). The centrality measures the importance of the selected theme, and density measures the development of the chosen theme. The graph is divided into four parts. Themes that appear on the lower left part are emerging or declining themes. These are new themes that can emerge to be better or drop from the research area. Themes that come under the lower right part of the thematic map are the basic or transversal themes. These themes represent low density but high centrality. Much research has been done on these themes.

The Upper left part represents high density but lower centrality, these themes are highly developed but isolated. The upper right part represents high density and high centrality. The themes in this part are a motor theme, which is developed and essential [53]. The size of the thematic map is to the factors that come under the theme.

Thematic map of figure 11 is constructed based on a full-time span from 2003 to 2020. We have used the top 400 keywords, but items shown in the clusters are set to the minimum frequency of 4 in ‘biblioshiny’ web software. The number of representative labels in each theme is set to 3. This has no link with the literature but represents the subjective judgment of the authors keeping in view the dynamics and best representation of coronavirus literature.

Table 9 is constructed from the clusters shown in figure 11. First cluster is represented by SARS and topics under this cluster are related to risk management and assessment, public health, infectious disease spread and outbreak such as [54]–[60], [24] and [61]. According to the thematic map, these themes come under the basic or transversal themes with high centrality and low density. There is much work in these themes, but it is difficult to find future direction from them because most of the topics are covered. The topics covered in these clusters are related to the Second research theme has some part in emerging themes and many parts in transversal theme. The cluster of the stated theme is represented by humans. Topics related to different characteristics of the virus-host are discussed here. Significant research has been done, and more is required, that is why this theme is converting from emerging to transversal. Research topics under this themes are related to the effect of infectious disease on descriptive of humans such as male, female, adults, old age, middle age, residents, foreigners and travelers such as discussed in [62]–[64], [16], [65], and [66]. This research theme cluster is represented by a communicable disease. It is an emerging theme in coronavirus literature. This theme is indicating the management and organization of communicable diseases with various methodologies [67]–[71] and [72].

Researchers can look this emerging theme from the dimension of communicable disease and public surveillance and role of public health administration [73]–[76], [17] and [18]. Moto themes are highly contributory themes because of high centrality and high density. In coronavirus literature theme with infection control as the representative qualified to be in that part of thematic map. Main dimensions of this theme are public health service, education about infectious disease. Education covers both professional and general public education. Psychological impact of infectious diseases and attitude towards pandemic outbreak. Researchers can understand this theme by going through [77]–[84] and [85].

Themes related to avian influenza is considered as highly developed yet isolated. The density is high, but centrality is low. There is a lot of potential in these themes and researcher can do much impactful work in these themes. Avian influenza is linked with topics related to health care personal, attitude towards pandemic outbreak and acquired immune deficiency syndrome. Theme directions are widely discussed in [86]–[91], [25], [92]–[94] and [95]

The themes represented by coronavirus is also highly developed and isolated. How are these respiratory and viral diseases affecting humans? This theme sees the link between coronavirus, disease outbreak, epidemic, gender and age [96]–[99] and [100]. Finally this theme covers latest computer simulated methods to identify various dynamics of infectious diseases [101]–[103], [42], [104] and [105].

This research stream can be linked with basic themes which are represented by coronavirus and researchers can develop a drug that can be helpful to curb the disease spread. The risk assessment identifies various risk factor involved in the spread of viral diseases. It can be controlled by public feedback. A significant amount of research can be done with public surveys. It can be linked with basic themes and check the risk tolerance, public reactions and preventions with respect to gender, age, and ethnicity. It adds to the body of knowledge and helps in infection control. Infection control is also part of the motor theme. Coronavirus is generated from animals so these themes should be linked with motor and basic themes as well. If research is conducted on emerging themes, then these themes can move to basic or motor themes in future.

#### 3) Thematic Evolution

**FIGURE 12.**
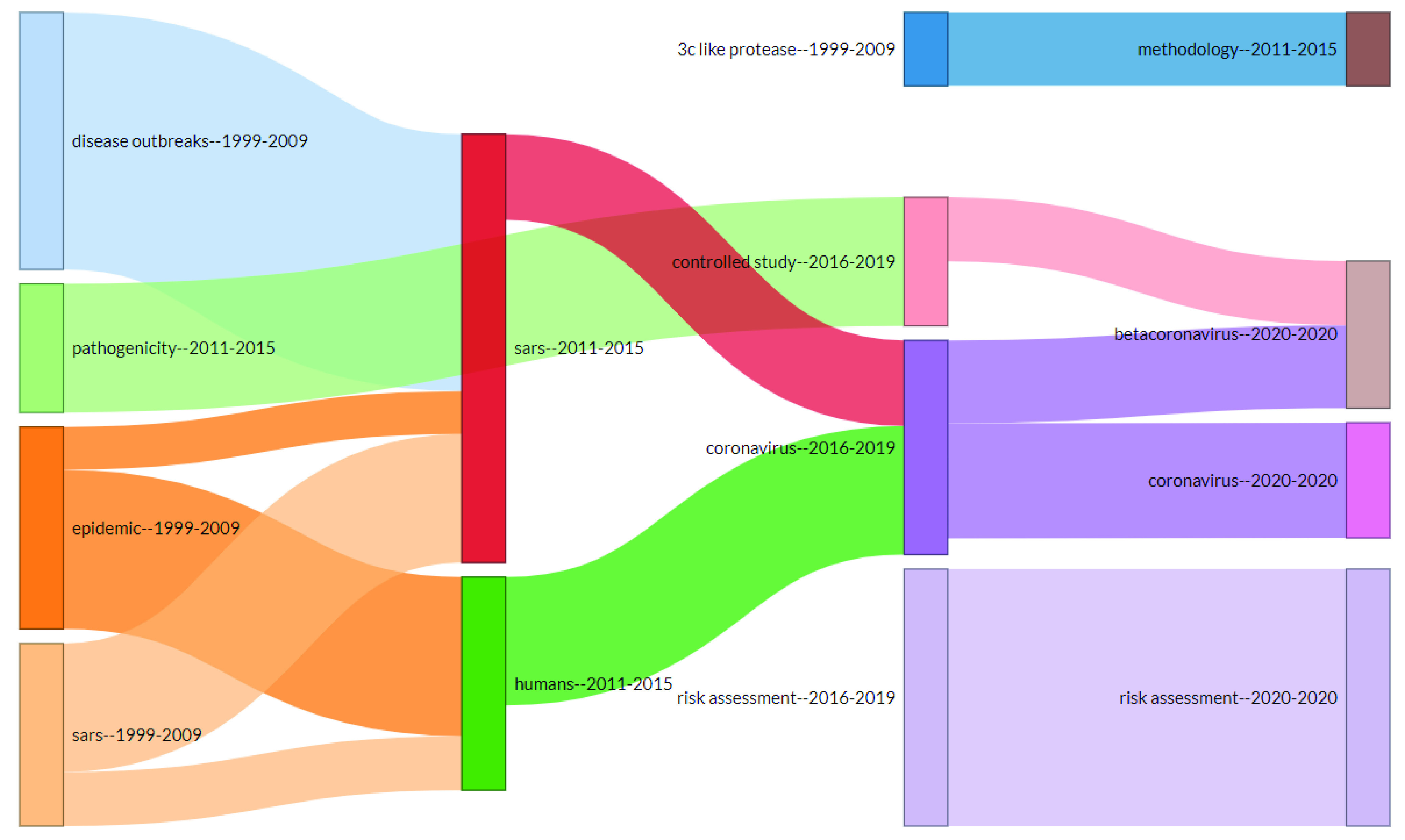
Thematic Evolution.

In addition to the thematic map there is thematic evolution (figure 12), that is showing the historical development of coronavirus literature. Using the keywords plus the thematic evolution depicts the history of themes and how these themes evolved. The thematic evolution is made using ‘biblioshiny’ and with four segments of time. This time segmentation is based on the subjective judgement of the authors keeping in view the better representation of thematic evolution. The first segment is from 1999 to 2010, the second segment is from 2009 to 2015, third is from 2015 to 2019 and last segment is from 2019 to 2020. Themes have evolved with time. As analyzed from 1999 to 2010 the coronavirus literature was about a disease outbreak, epidemic, SARS. From 2011 to 2015 research in SARS got increased and topics related to disease outbreaks evolved into SARS literature. Furthermore, part of epidemic literature evolved into SARS and other parts into effects on humans. Pathogenicity was introduced during the period 2011 to 2015 but evolved into controlled study literature from 2016 to 2019. 3c like protease, was a drug had some success for SARS patients. Studies on the topic were available from 1999 till 2009 but after that it got evolved with other methodologies for protection and disease control.

From 2016 till date topic related to risk assessment are under observation. Part of SARS literature has been used in coronavirus literature and humans’ effect is also studied in coronavirus pandemic literature.

## VI. Conclusions

This study has found significant influential aspects of coronavirus literature. These influential aspects provide implication for core future research. The study finds that ‘Morbidity and Mortality Weekly Report’, ‘Social Science and Medicine’ and ‘Health Security’ are top three journals with highest impact in coronavirus literature. There are 23 core journals according to Bradford law. Tablan *et al.* [21] is the core jounral article with most global citations, who reported the guidelines that are formed to decrease the incidence of pneumonia and other SARS related infections. ‘SARS’ is the word that is most frequently used in titles, author keywords and keywords plus and ‘Health’ in abstract. K.W. Chau and D.C.W. Ho are the authors having the maximum impact according to coronavirus literature. University of Hong Kong is the most relevant affiliation for publishing coronavirus literature. USA has most publications but articles from Hong Kong have most citations. USA is also number one in terms of the corresponding authorship, with correspondence in 64 articles (48 SCP, 16 MCP). Hong Kong and Canada come on second and third. The main collaboration of research is in between the USA and China. The USA also collaborated with Korea, Canada, Taiwan, and Hong Kong.

Conceptual structure from ‘biblioshiny’ R-package has provided key research streams and themes. We have identified four core research streams in coronavirus literature by using co-occurrence network. These research streams are; ‘Social and economic effects of epidemic disease’, ‘Infectious disease calamities and control’, ‘Outbreak of COVID 19’ and ‘Infectious diseases and the role of international organizations’. Combining these research streams will address many issues in the times of pandemic attack.

Furthermore, in conceptual structure, the study has deployed thematic map to put the themes and subthemes on graph and divides them into four parts (dropping or emerging themes, basic themes, highly developed and isolated themes, motor themes). Basic or transversal themes are represented by SARS, risk assessment and management, public health, infectious disease transmission and control and role of world health organization. Moreover, impact of infectious disease on humans and various demographics of humans are also basic themes. Methodologies related to communicable disease and public surveillance; health administrations are emerging themes. Public health services and education for infection control and psychological impact on behaviors and attitudes created by infectious diseases are motor themes. Highly developed but isolated themes of corona literature are topics related to Avian influenza.

### A. Future Agendas

As we have reviewed the coronavirus literature thoroughly by looking at various influential aspects and conceptual framework. We can set forth some directions for researchers, doctors, policy makers to investigate the right aspects for answers of alarming issues.

1) Overall limited studies are there on coronavirus outbreak in social sciences. More research is required.
2) Availability of funds for core journals such as the sources found under zone 1 by using Bradford law can create quality future research.
3) Collaboration with core authors, affiliations, and understanding the globally cited articles will create quality future research.
4) According to the influential structure USA and Hong Kong are the coronavirus research hub in social sciences. These two research hubs should collaborate with health and research departments of underdeveloped countries where COVID-19 effects are limited. They should study these countries social habits and vaccine routines. ‘
5) Very limited collaboration among countries has been observed. More collaboration for infectious disease policy and control is required in social sciences fields.

The conceptual framework has cleared some themes for research directions. These directions can be explored by using the following points

6) In the co-occurrence network there are various themes identified with various colors red cluster is the basic cluster relating to public health, uncertainty, epidemic and economy. On the other hand, publications of purple cluster are related to infectious disease and role of international organizations. The link between a red cluster and purple cluster can create strategies for international health organizations to curb the spread of infectious disease and suggest economic development plan under pandemic situations. Similarly, red cluster publications can be linked with blue to identify and study risk factors that are responsible for disease spread. Blue cluster with green can generate studies to identify resilience strategies and crisis management from SARS and MERS related studies and apply them to outbreak of COVID 19.
7) Methodologies and management of Communicable diseases are the emerging theme. Future research is required in this research field.
8) SARS is the old virus in the family of coronaviruses, but the findings related to SARS can be used to identify remedies for novel coronavirus.
9) For theme identification we have used keyword plus. There should be a software that can take essential terms from all sources such as author’s keywords, keywords plus, abstract and title and produce the final form of keywords for the conceptual framework. Studies for such software methodology are required

### B. Limitations of the Study

The main limitation of the study is that there is very limited literature available on coronavirus in social science, economics and business field. More research is required to tackle the needs of the economy in a pandemic situation. Furthermore, Due to the limited number of author’s keywords, this study only used keywords plus for development of various themes.
